# Injury profile, prevalence and risk factors among South African Para-athletes

**DOI:** 10.4102/ajod.v15i0.1840

**Published:** 2026-03-12

**Authors:** Siyabonga H. Kunene

**Affiliations:** 1 Department of Physiotherapy, Faculty of Health Sciences, University of the Witwatersrand, Johannesburg, South Africa; 2 Department of Wits Sports and Health, Faculty of Health Sciences, University of the Witwatersrand, Johannesburg, South Africa

**Keywords:** Para-athletes, sports injuries, injury prevalence, risk factors, rehabilitation and prevention, South Africa

## Abstract

**Background:**

Para-athletes are at heightened risk of sports-related injuries due to sport-specific demands, disability types and environmental exposures. Despite global research, limited data exist on injury profiles in low-resource settings such as South Africa.

**Objectives:**

To profile injuries, determine their prevalence and identify associated risk factors among South African Para-athletes.

**Method:**

A cross-sectional study was conducted among 86 South African Para-athletes (response rate: 74.14%) using a self-developed questionnaire. Data on demographics, disability type, sporting history, injury history and risk factors were analysed using descriptive statistics and logistic regression.

**Results:**

Among 86 Para-athletes (median age 27 years), limb deficiency was the most common impairment (50%) and athletics (44%) the leading sport. Athletes trained 5.3 sessions per week and competed 3.4 days/quarter; adapted environments were significantly more common in men (67% vs. 33%, *p* = 0.015). Median injuries over 12 months were three, mainly in the lower quadrant (74%, *p* = 0.617), with strains (34%) and sprains (24%) most frequent. Multivariable analysis showed that swimming (odds ratio [OR] = 0.05, *p* = 0.010) and strength training (OR = 0.05, *p* = 0.002) were strong protective factors against injury. In contrast, skipping a warm-up during training significantly increased injury risk (OR = 3.2, 95% confidence interval [CI]: 1.3–7.9, *p* = 0.011), with athletes who did not warm up being more than three times as likely to sustain an injury compared with those who performed a warm-up.

**Conclusion:**

South African Para-athletes experience a substantial injury burden, highlighting the need for tailored prevention strategies and improved access to rehabilitation and education.

**Contribution:**

This study provides the first comprehensive injury profile of South African Para-athletes, informing inclusive, evidence-based interventions.

## Introduction

Athletes living with disabilities face various challenges, including high prevalence of injuries with specific risks varying by sports, disability type and demographics. Musculoskeletal injuries are most common types, particularly shoulder- and elbow-related injuries. The prevalence of these musculoskeletal injuries is reported to be approximately 40.8% (Pinheiro et al. 2021). The incidence rate of these injuries has been reported to be about 14.3 injuries per 1000 athlete-days. Upper extremity (especially shoulder and elbow) is usually the most injured part of the body in all Para-athletes compared to able-bodied athletes (Garcia-Carrillo et al. 2024; Pinheiro et al. 2021, 2024; Tuakli-Wosornu et al. 2018). These injuries included muscle strains, ligament sprains, contusions and epidermal abrasions.

Seated Para-athletes report with upper limb injuries, whereas those who ambulate sustain lower limb injuries (Fagher & Lexell 2014; Tuakli-Wosornu et al. 2018). Para-athletes have reported with minor soft tissue injuries as seen in injury patterns seen in able-bodied athletes. The following summer sports showed highest risks of injuries: powerlifting, football five-aside, wheelchair fencing, goalball and wheelchair rugby. Winter sports with highest risks of injuries included ice hockey, snowboarding and alpine skiing. Varying prevalence rates are shown in specific sports, but, Para powerlifting seems to be reported as having the highest injuries due to the high intensity and increased risks of the nature of sport (Pinheiro et al. 2024). The incidence of Para powerlifting is estimated at 15.6 per 1000 athlete-days (Ona Ayala et al. 2019).

In other specific sports like Para throwers, Garcia-Carrillo et al. (2024) reported a 12-month injury prevalence of 40%. For adaptive sports athletes, the prevalence ranges from 0% in adaptive rowing to about 60% in sled hockey according to Soo Hoo, Latzka and Harrast (2019).

Para-athletes can present with specific injuries including bone stress injuries which are reported in 9.2% of Para-athletes, with common sites being the metatarsals and hand or wrist (Tenforde et al. 2019). Overuse injuries, including muscle strains and tendinopathies are also common, especially in sports requiring repetitive movements (Tuakli-Wosornu et al. 2018). Acute injuries turn to peak during competition times as seen in able-bodied athletes.

Different Para sporting codes present with unique risks based on their nature of sports, disability type, training and experience and various demographics. For example, athletes using upper limb (e.g. wheelchair athletes) will be more prone to shoulder girdle injuries and osteoporotic fractures, while those ambulating (e.g. cerebral palsy athletes) will be more prone to knee, foot and ankle injuries (Sacks et al. 2022; Weijer et al. 2024). Another example is Para powerlifting that will have higher prevalence of injuries compared to swimming (Pinheiro et al. 2024).

Training and competition factors may also contribute to Para-athletes sustaining injuries. Sudden change in training intensity and competition periods is linked to higher rates of injuries (Busch et al. 2025). Overuse injuries are most common, especially during training, while acute injuries happen more during competition periods (Garcia-Carrillo et al. 2024).

Gender and sports experience is another factor that has been linked with risks of injuries in Para sports (Busch et al. 2025). Women have higher risks of injuries, compared to men. Athletes who participate at an elite level with more than 5 years are usually presenting with lower risks of injuries.

Although recent literature has examined injuries among Para-athletes across various contexts, a significant gap remains in understanding injuries and associated risks, particularly in low socioeconomic settings such as South Africa. Research focusing on Para-athletes in the Southern African region is notably scarce. In countries like South Africa, Para-athletes continue to face numerous challenges, including the absence of comprehensive injury profiles, persistent discrimination and stigma, limited opportunities, poor access to essential services and resources, inadequate facilities and a general lack of awareness regarding injury prevention, management and rehabilitation (Kunene 2023, 2025).

There is an urgent need to advance disability inclusion in low socioeconomic countries like South Africa, beginning with a thorough investigation of the existing challenges. Understanding these challenges is a critical first step towards developing effective strategies to address them. This study aims to fill this gap by profiling injuries and determining their prevalence and associated risk factors among Para-athletes in South Africa. To the best of the author’s knowledge, no prior study has comprehensively addressed this issue. Generating this knowledge is essential for mitigating injury risks and enhancing prevention, treatment and rehabilitation efforts. Tailored interventions and systematic monitoring are vital to reducing injury incidence and promoting the overall health and well-being of Para-athletes.

## Research methods and design

### Study design

This study employed a cross-sectional design, an observational research approach in which data were collected at a single point in time. This design is appropriate for identifying patterns and associations within a defined population without manipulating variables.

### Participants and sampling

The population consisted of South African Para-athletes. Eligibility criteria included being 18 years or older and having no intellectual disabilities. Participants with physical disabilities were included, encompassing individuals with visual impairments, neurological impairments, dwarfism and limb amputations. Only athletes who could read, understand and respond in English, either verbally or in writing, with or without assistance, were eligible to participate. According to South African Sports Association for Physically Disabled (SASAPD), English proficiency is common among Para-athletes in South Africa. The estimated total population size for this study was approximately 116 athletes. A sample size of 86 was determined using a Raosoft statistical tool, with a confidence level set at 95%, margin error at 5% and response distribution at 50%.

### Data collection tool

This cross-sectional study utilised a self-developed questionnaire consisting of five sections designed to collect data on participants’ demographics, type of disability, sporting history, injury history and associated risk factors. The questionnaire was written in English and tailored to a readability level appropriate for individuals aged 13–15 years, with a Flesch Reading Ease score between 60 and 70, corresponding to an 8th – 9th grade reading level.

The content of the questionnaire was informed by current literature in the fields of sports medicine and rehabilitation. To establish content and face validity, the questionnaire was reviewed by five experienced experts (over 10 years of experience working and researching in the field) in sports and exercise physiotherapy, who provided feedback on its clarity, relevance and comprehensiveness. A pilot study involving nine participants (representing 10% of the intended sample size) was conducted to further validate the tool. The pilot study revealed no areas for improvement in the developed questionnaire. Data from the pilot study were subsequently included in the main analysis.

The final version of the questionnaire was administered using the Research Electronic Data Capture (REDCap) system, which served as the primary platform for distribution and response collection. For participants who were unable to access the online version, printed copies were provided and completed in person.

### Recruitment and data collection

Participants were recruited through SASAPD. An invitation email was sent to the association, requesting distribution of the study details to eligible athletes. The email included an information sheet and a REDCap link to access the online questionnaire. Interested participants completed a self-administered questionnaire, which took approximately 20–30 min.

In addition to the online format, face-to-face meetings were held with eligible participants who preferred to complete a printed version of the questionnaire. Written informed consent was obtained from each participant prior to data collection.

To enhance participation, weekly reminders were disseminated via SASAPD as well as through sports federations and managers or coaches of various sporting codes. The data collection period lasted 3 weeks.

### Data analysis

Upon completion of the questionnaire, either via REDCap or the printed version, the data were exported and captured into Microsoft Excel for preliminary cleaning. This process involved coding participants’ responses and preparing the dataset for statistical analysis. The cleaned data were then imported into IBM SPSS Statistics for analysis. Categorical data were reported using frequencies and percentages (Kirkwood & Sterne 2003). Quantitative data that were normally distributed were described using means and standard deviations. Data that were not normally distributed were described using medians and interquartile ranges (IQR). Normality was examined using a combination of graphical methods as well as the formal Shapiro–Wilk test (Razali & Wah 2011) at the 5% significance level.

To examine factors associated with injuries, univariable and multivariable logistic regression models were considered (Tabachnick & Fidell 2019). Model specification for the multivariable logistic regression was carried out using the Bayesian Information Criterion (Burnham & Anderson 2002), moving from a saturated model to a parsimonious model.

### Ethical considerations

This study received ethical clearance from the University of the Witwatersrand Human Research Ethics Committee (Clearance No. M220120). In addition, written permission was secured from the SASAPD. All potential participants received detailed information about the study and provided written informed consent before participating. To ensure confidentiality, the questionnaire did not collect any personally identifiable information. Instead, unique codes were assigned to each participant for data analysis purposes. All collected data were securely stored in a password-protected online drive, accessible only to the principal investigator and the designated statistician.

## Results

### Demographics

A total of 86 Para-athletes participated in the study, yielding a response rate of 74.14%. The majority of participants were black people (45.4%), with a gender distribution of 45 men and 41 women (Table 1). The mean age of participants was 27 years. Most athletes (50%) presented with limb deficiencies. Athletics (44.2%), swimming (23.3%) and cerebral palsy (CP) football (12.8%) were the most common sports. Median sporting experience was 6 years (IQR: 4–7).

**TABLE 1:** Demographics and sports history and exposure characteristics by sex (*N* = 86).

Variable	All (*n* = 86)		Male (*n* = 45)		Female (*n* = 41)	
	*n*	%	*n*	%	*n*	%
Age (years)†	27	-	27	-	27	-
**Race**						
Black people	39	45.4	23	51.1	16	39.0
White people	35	40.7	16	35.6	19	46.3
Indian people	4	5.7	3	6.7	1	2.4
Coloured people	6	7.0	3	6.7	3	7.3
Asian people	2	2.3	0	0.0	2	4.9
**Side dominance**						
Right	53	61.6	28	62.2	25	61.5
Left	27	31.4	14	31.1	13	31.4
Ambidextrous	6	7.0	3	6.7	3	7.3
**Disability type**						
Short stature	11	12.8	6	13.3	5	12.2
Limb deficiency	43	50.0	23	51.1	20	48.8
Impaired joint ROM	4	4.7	2	4.4	2	4.9
Impaired muscle power	5	5.8	1	2.2	4	9.8
Hypertonia	9	10.5	6	13.3	3	7.3
Ataxia	3	3.5	1	2.2	2	4.9
Athetosis	3	3.5	1	2.2	2	4.9
Visual impairment	8	9.3	5	11.1	3	7.3
**Sporting code**						
Athletics	38	44.2	21	46.7	17	41.5
CP football	11	12.8	8	17.8	3	7.3
Swimming	20	23.3	6	13.3	14	34.1
Goalball	3	3.5	2	4.4	1	2.4
Boccia	2	2.3	1	2.2	1	2.4
Powerlifting	2	2.3	2	4.4	0	0.0
Paracycling	2	2.3	2	4.4	0	0.0
Judo	1	1.2	1	2.2	0	0.0
Wheelchair basketball	7	8.1	2	4.4	5	12.2
Sporting Experience‡	6	-	5	-	6	-

RON, range of motion; CP, cerebral palsy.

† , Age median (IQR) = 22–33;

‡ , Sporting experience median (IQR) = 4–7.

### Sport participation

Table 2 shows that participants had an average of 5.3 weekly training sessions and a median duration of 60 min per session, showing no sex-based differences. Cross-training (40.7%) and strength training (38.4%) were common, and most participants (76.7%) took rest days and competed about 3.4 days/month, with no significant sex differences. Training mainly occurred on track (49%) and field (37%), but more men trained in adapted environments (67%) than women (33%) (*p* = 0.015). Adapted environments were significantly more common among men (67%) than women (33%) (*p* = 0.015). Indoor and outdoor training were equally distributed (50% each), and 71% of athletes used specific equipment, with no significant gender differences.

**TABLE 2:** Sporting participation and exposure characteristics by sex (*N* = 86).

Variable	All (*n* = 86)			Male (*n* = 45)			Female (*n* = 41)			*p*-value
	Value	*n*	%	Value	*n*	%	Value	*n*	%	
**Training sessions per week**	-	-	-	-	-	-	-	-	-	0.644
Mean	5.3	-	-	5.3	-	-	5.2	-	-	-
s.d.	1.0	-	-	1.0	-	-	1.0	-	-	-
**Duration per session (minutes)**	-	-	-	-	-	-	-	-	-	0.812
Median	60	-	-	60	-	-	60	-	-	-
IQR	30–120	-	-	30–120	-	-	30–120	-	-	-
**Cross-training**	-	-	-	-	-	-	-	-	-	0.425
Yes	-	35	40.7	-	16	45.7	-	19	54.3	-
No	-	51	59.3	-	29	56.9	-	22	43.1	-
**Strength training**	-	-	-	-	-	-	-	-	-	0.905
Yes	-	33	38.4	-	17	51.5	-	16	48.5	-
No	-	53	61.6	-	28	52.8	-	25	47.2	-
**Rest days**	-	-	-	-	-	-	-	-	-	0.208
Yes	-	66	76.7	-	37	56.1	-	29	43.9	-
No	-	20	23.3	-	8	40.0	-	12	60.0	-
**Competition days per quarter**	-	-	-	-	-	-	-	-	-	0.649
Mean	3.4	-	-	3.5	-	-	3.4	-	-	-
s.d.	1.2	-	-	1.3	-	-	1.2	-	-	-
**Type of environment**	-	-	-	-	-	-	-	-	-	0.596
Field	-	32	37.0	-	19	59.0	-	13	41.0	-
Track	-	42	49.0	-	20	48.0	-	22	52.0	-
Other (Gym, swimming, indoor court)	-	12	14.0	-	6	50.0	-	6	50.0	-
**Environment adapted**										0.015*
Yes	-	39	46.0	-	26	67.0	-	13	33.0	-
No	-	47	54.0	-	19	40.0	-	28	60.0	-
**Indoor or outdoor**	-	-	-	-	-	-	-	-	-	0.131
Indoor	-	43	50.0	-	19	44.0	-	24	56.0	-
Outdoor	-	43	50.0	-	26	60.0	-	17	40.0	-
**Use specific equipment**	-	-	-	-	-	-	-	-	-	0.165
Yes	-	61	71.0	-	29	48.0	-	32	52.0	-
No	-	25	29.0	-	16	64.0	-	9	36.0	-
**Equipment condition**	-	-	-	-	-	-	-	-	-	0.474
Fairly new	-	23	26.7	-	13	56.5	-	10	43.5	-
Not new, but good condition	-	35	40.7	-	20	57.1	-	15	42.9	-
Worn out/past expected time	-	28	32.6	-	12	42.9	-	16	57.1	-
**Equipment replacement**	-	-	-	-	-	-	-	-	-	0.805
Replaced when due	-	40	46.5	-	22	55.0	-	18	45.0	-
Replaced after due date	-	46	53.5	-	23	50.0	-	23	50.0	-
**Hot weather**	-	-	-	-	-	-	-	-	-	0.250
Not affected	-	5	5.8	-	1	20.0	-	4	(80.0)	-
Slightly difficult	-	31	36.0	-	17	54.8	-	14	(45.2)	-
Moderately difficult	-	41	47.7	-	24	58.5	-	17	(41.5)	-
Very difficult	-	9	10.5	-	3	33.3	-	6	(66.7)	-
**Cold weather**	-	-	-	-	-	-	-	-	-	0.990
Not affected	-	15	17.4	-	8	53.3	-	7	46.7	-
Slightly difficult	-	39	45.4	-	21	53.8	-	18	46.2	-
Moderately difficult	-	22	25.6	-	11	50.0	-	11	50.0	-
Very difficult	-	10	11.6	-	5	50.0	-	5	50.0	-
**Warm-up during training**	-	-	-	-	-	-	-	-	-	0.382
Yes	-	42	49.0	-	24	57.1	-	18	42.9	-
No	-	44	51.0	-	21	47.7	-	23	52.3	-
**Warm-up during competition**	-	-	-	-	-	-	-	-	-	0.909
Yes	-	54	63.0	-	28	51.9	-	26	48.1	-
No	-	32	37.0	-	17	53.1	-	15	46.9	-
**Cool-down during training**	-	-	-	-	-	-	-	-	-	0.218
Yes	-	34	39.5	-	15	44.1	-	19	55.9	-
No	-	52	60.5	-	30	57.7	-	22	42.3	-
**Cool-down during games**	-	-	-	-	-	-	-	-	-	0.318
Yes	-	21	24.4	-	9	42.9	-	12	57.1	-
No	-	65	75.6	-	36	55.4	-	29	44.6	-

s.d., standard deviation; IQR, interquartile range.

* , significant at 0.05.

Equipment condition was generally good, with 40.7% reporting ‘not new but good condition’. Regarding equipment replacement, 46.5% of athletes reported replacing equipment when due, while 53.5% delayed replacement beyond the recommended time frame. Among men, 55% replaced equipment on time, compared to 45% of women, whereas late replacement was reported by 50% of men and 50% of women.

Environmental challenges were notable: 47.7% of athletes found hot weather moderately difficult, while 36% reported slight difficulty. Cold weather was less problematic, with 45.4% reporting slight difficulty and 25.6% moderate difficulty. Warm-up practices were inconsistent; 49% warmed up during training and 63% during competition, while cool-down was less common (39.5% during training and 24.4% during games). None of these differences reached statistical significance.

### Injury history and characteristics

The median number of injuries reported in the past 12 months was three (IQR: 2–3) overall, with men experiencing slightly more injuries (three [IQR: 2–4]) compared to women (two [IQR: 2–3]), although this difference was not statistically significant (*p* = 0.279) (Table 3). Most injuries occurred in the lower quadrant (back, hip, thigh, knee, leg, ankle and foot), accounting for 74% of all injuries, while 26% were in the upper quadrant (head, neck, shoulder, elbow, arm, wrist and hand) (*p* = 0.617).

**TABLE 3:** Injury history and exposure characteristics (*N* = 86).

Variable	All (*n* = 86)			Male (*n* = 45)			Female (*n* = 41)			*p*-value
	Value	*n*	%	Value	*n*	%	Value	*n*	%	
**Number of injuries in past 12 months**	-	-	-	-	-	-	-	-	-	0.279
Median	3	-	-	3	-	-	2	-	-	-
IQR	2–3	-	-	2–4	-	-	2–3	-	-	-
**Injury location**	-	-	-	-	-	-	-	-	-	0.617
Upper quadrant (head, neck, shoulder, elbow, arm, wrist, hand)	-	22	26.0	-	10	45.0	-	12	55.0	-
Lower quadrant (back, hip, thigh, knee, leg, ankle, foot)	-	64	74.0	-	35	55.0	-	29	45.0	-
**Most troubling injury type**	-	-	-	-	-	-	-	-	-	0.313
Sprain	-	21	24.4	-	8	17.8	-	13	31.7	-
Strain	-	29	33.7	-	18	40.0	-	11	26.8	-
Fracture	-	7	8.1	-	2	4.4	-	5	12.2	-
Joint dislocation	-	6	7.0	-	5	11.1	-	1	2.4	-
Joint pain (without fracture)	-	9	10.5	-	5	11.1	-	4	9.8	-
Skin bruise	-	9	10.5	-	5	11.1	-	4	9.8	-
Concussion	-	5	5.8	-	2	4.4	-	3	7.3	-
**Medical or rehab received**	-	-	-	-	-	-	-	-	-	1.000
Yes	-	39	45.4	-	20	44.4	-	19	46.3	-
No	-	47	54.6	-	25	55.6	-	22	53.7	-
**Injuries during training**	-	-	-	-	-	-	-	-	-	0.453
Upper quadrant (head, neck, shoulder, elbow, arm, wrist, hand)	-	19	22.0	-	8	42.0	-	11	58.0	-
Lower quadrant (back, hip, thigh, knee, leg, ankle, foot)	-	67	78.0	-	37	55.0	-	30	45.0	-
**Injuries during competition**	-	-	-	-	-	-	-	-	-	1.000
Upper quadrant (head, neck, shoulder, elbow, arm, wrist, hand)	-	11	13.0	-	6	55.0	-	5	45.0	-
Lower quadrant (back, hip, thigh, knee, leg, ankle, foot)	-	75	87.0	-	39	52.0	-	36	48.0	-
**Injury prevention education**	-	-	-	-	-	-	-	-	-	0.721
Yes	-	35	40.7	-	17	37.8	-	18	43.9	-
No	-	51	59.3	-	28	62.2	-	23	56.1	-

IQR, interquartile range.

The most troubling injury types were strains (33.7%) and sprains (24.4%), followed by fractures (8.1%), joint dislocations (7.0%), joint pain without fracture (10.5%), skin bruises (10.5%) and concussions (5.8%). There were no significant gender differences in injury-type distribution (*p* = 0.313).

Regarding medical or rehabilitation care, 45.4% of athletes reported to having received treatment for their injuries, while 54.6% did not (*p* = 1.000). Injuries during training were more frequent than during competition, with 78% occurring in the lower quadrant and 22% in the upper quadrant during training (*p* = 0.453). Similarly, during competition, 87% of injuries affected the lower quadrant and 13% the upper quadrant (*p* = 1.000).

Injury prevention education was reported by 40.7% of athletes, while 59.3% had not received such education, with no significant gender difference (*p* = 0.721).

### Factors associated with injuries (univariable analysis)

Univariable logistic regression analysis identified both protective and risk factors for injury among athletes. Swimming was a significant protective factor (OR = 0.3, 95% CI: 0.1–0.9, *p* = 0.034) (Table 4), with swimmers being approximately 70% less likely to sustain injuries compared to non-swimmers. Similarly, engaging in strength training showed a strong protective effect (OR = 0.3, 95% CI: 0.1–0.7, *p* = 0.011), reducing injury risk by about 70%. In contrast, skipping warm-up during training markedly increased injury risk (OR = 3.2, 95% CI: 1.3–7.9, *p* = 0.011), making athletes more than three times as likely to be injured compared to those who warmed up. Additionally, athletes who reported moderate difficulty training in cold weather had nearly threefold higher odds of injury compared to those not affected (OR = 2.8, 95% CI: 1.2–6.5, *p* = 0.015).

**TABLE 4:** Univariable logistic regression model for factors associated with injuries (*N* = 86).

Variable	OR	95% CI	*p*-value
Gender (Ref: Male)	1.2	0.5–2.8	0.617
Age (years)	1.0	0.9–1.1	0.812
Sporting experience (years)	1.0	0.9–1.1	0.745
Training sessions per week	1.1	0.9–1.3	0.312
Duration per session (minutes)	1.0	0.9–1.1	0.654
Swimming (yes vs. no)	0.3	0.1–0.9	0.034*
Strength training (yes vs. no)	0.3	0.1–0.7	0.011**
Warm-up during training (no vs. yes)	3.2	1.3–7.9	0.011**
Medical or rehab received (yes vs. no)	1.1	0.5–2.4	0.812
CP football (yes vs. no)	0.3	0.07–1.2	0.089
Cold weather moderately difficult (vs. not affected)	2.8	1.2–6.5	0.015*

OR, odds ratio; CI, confidence interval; CP, cerebral palsy.

* , Significant (*p* < 0.05);

** , Very significant (*p* < 0.01).

### Factors associated with injuries (multivariable analysis)

Multivariable analysis revealed several strong protective and risk factors for injury. Swimming was highly protective (OR = 0.05, 95% CI: 0.005–0.5, *p* = 0.010) (Table 5), with swimmers being 95% less likely to sustain injuries. Strength training demonstrated a similar effect (OR = 0.05, 95% CI: 0.01–0.3, *p* = 0.002), substantially reducing injury risk. Conversely, increased competition days per month was a significant risk factor (OR = 2.7, 95% CI: 1.1–6.8, *p* = 0.036), nearly tripling injury odds. Equipment in good condition offered protection (OR = 0.1, 95% CI: 0.02–0.8, *p* = 0.030), while athletes reporting extreme difficulty in hot weather showed markedly lower injury risk (OR = 0.002, 95% CI: 0.000005–0.6, *p* = 0.031), possibly due to adaptive strategies. Similarly, moderate difficulty in cold weather was associated with reduced injury odds (OR = 0.1, 95% CI: 0.04–0.7, *p* = 0.023). In contrast, skipping cool-down during training significantly increased injury risk eightfold (OR = 8.2, 95% CI: 1.2–54.1, *p* = 0.029).

**TABLE 5:** Multivariable logistic regression model for factors associated with injuries (*N* = 86).

Variable	OR	95% CI	*p*-value
Swimming (yes vs. no)	0.05	0.005–0.5	0.010**
Strength training (no vs. yes)	0.05	0.01–0.3	0.002***
Competition days per month	2.7	1.1–6.8	0.036*
Equipment condition (not new, but still good)	0.1	0.02–0.8	0.030*
Hot weather very difficult (vs. not affected)	0.002	0.000005–0.6	0.031*
Cold weather moderately difficult (vs. not affected)	0.1	0.04–0.7	0.023*
Cool down during training (no vs. yes)	8.2	1.2–54.1	0.029*

OR, odds ratio; CI, confidence interval.

* , Significant (*p* < 0.05);

** , Very significant (*p* < 0.01);

*** , Highly significant (*p* < 0.001).

## Discussion

This discussion highlights key findings from the study on sports-related injuries and risk factors among South African Para-athletes. A high median of three sports-related injuries per athlete per year indicates a substantial injury burden in this population. The lower quadrant (back, hip, thigh, knee, leg and ankle) were consistently the most affected body regions during both training and competition, while strains and sprains emerged as the most common injury types. These findings suggest potential biomechanical vulnerabilities and training load issues and are consistent with global and South African literature. As noted in the introduction, Para-athletes worldwide experience a high prevalence of musculoskeletal injuries, estimated at 40.8%, with strains and sprains being predominant (Garcia-Carrillo et al. 2024; Tenforde et al. 2019). Although upper limb injuries are frequently reported in Para sport (Pinheiro et al. 2021; Tuakli-Wosornu et al. 2018), the predominance of lower limb injuries in this study may be attributed to the high representation of ambulant athletes participating in athletics.

No significant differences were observed between men and women athletes in terms of injury prevalence, location, type or exposure variables. This finding supports the view that injury risk in Para sport is more strongly influenced by sport-specific demands and individual characteristics than by sex. While sex-based differences in injury patterns are well-documented in able-bodied athletes (Mallorquín et al. 2025), they may not be as prominent in Para sport due to the complex interplay of disability type, sport classification and functional capacity. Injury risk in this population is multifactorial, with contributing factors including the nature of the sport, individual biomechanics and coping strategies, rather than biological sex alone.

A considerable proportion of athletes did not engage in warm-up or cool-down routines, particularly during training, and this was significantly associated with increased injury risk. Warm-ups are widely recognised for enhancing performance and reducing injury risk (Sople & Wilcox 2025), while cool-downs play a vital role in recovery and injury prevention (Gremion 2005). Despite their known benefits, there is limited sport-specific evidence on these practices within Para sport contexts. The findings contribute valuable insights, reinforcing general knowledge about the protective effects of warm-ups and cool-downs and highlighting the increased injury risk associated with their absence. This underscores the need for targeted education and structured implementation of these practices in Para sport training programmes.

Strength training emerged as a protective factor, reinforcing its role in injury prevention and functional resilience in Para-athletes. This factor is also widely accepted as beneficial for improving performance and lowering the risks of injuries in athletes in general (Chen et al. 2025). This makes strength training a crucial component of athletes training regimen.

Several environmental and equipment-related factors emerged as important considerations in this study, particularly weather conditions during training and competition and the quality of sports equipment. These factors are critical in managing injury risk among Para-athletes. Adverse weather conditions, both hot and cold, were significantly associated with injury occurrence, underscoring the need for climate-adaptive training strategies. Additionally, the condition of sports equipment played a protective role, with athletes using equipment that was not new but still in good condition experiencing fewer injuries. These findings highlight the importance of maintaining equipment quality and tailoring training environments to accommodate both climatic and functional needs of Para-athletes.

Fewer than half of the athletes received medical or rehabilitation support or injury prevention education, indicating a gap in athlete care and knowledge dissemination. This presents an opportunity for federations and support teams to enhance education, access to care and structured prevention programmes. Poor injury rehabilitation poses a risk to athletes, potentially leading to further injuries and long-term health issues (Cook & Finch 2011).

The findings underscore the need for standardised injury surveillance, preventive training protocols and inclusive access to adapted environments and equipment. Tailored interventions should consider sport type, training practices and environmental exposures to reduce injury risk.

## Conclusion

This study highlights a significant burden of sports-related injuries among South African Para-athletes, with lower limb injuries, particularly strains and sprains, being most prevalent. The findings showed a multifactorial nature of injury risk in this population, shaped more by sport-specific demands, individual biomechanics and environmental conditions than by sex. The absence of warm-up and cool-down routines, limited access to medical support and inadequate injury prevention education were all associated with increased injury risk, while strength training and well-maintained equipment emerged as protective factors. These insights emphasise the urgent need for tailored injury prevention strategies, climate-adaptive training environments and improved access to rehabilitation and education. Implementing standardised injury surveillance and inclusive, evidence-based interventions will be critical to safeguarding the health and performance of Para-athletes in South Africa and beyond.
